# Human atlastin GTPases mediate differentiated fusion of endoplasmic reticulum membranes

**DOI:** 10.1007/s13238-015-0139-3

**Published:** 2015-03-17

**Authors:** Xiaoyu Hu, Fuyun Wu, Sha Sun, Wenying Yu, Junjie Hu

**Affiliations:** 1 Tianjin Key Laboratory of Protein Sciences, Department of Genetics and Cell Biology, College of Life Sciences, Nankai University, Tianjin, 300071 China; 2 National Laboratory of Biomacromolecules, Institute of Biophysics, Chinese Academy of Sciences, Beijing, 100101 China

**Dear Editor,**

In eukaryotic cells, the endoplasmic reticulum (ER) is a continuous membrane system with interconnected tubules and sheets. The formation of a typical network requires constant fusion between ER membranes. A class of membrane-bound dynamin-like GTPases called atlastins (ATLs) has been identified as mediating ER fusion (Hu et al., 2009; Orso et al., 2009). The depletion of ATL results in an unbranched ER phenotype (Rismanchi et al., 2008; Hu et al., 2009), indicating a lack of interconnections among ER membranes. Antibodies against ATL block ER network formation *in vitro *when using Xenopus egg membrane extracts (Hu et al., 2009). The fusogenic activity of ATL is also observed when purified full-length Drosophila ATL is reconstituted into proteoliposomes, with the fusion of these vesicles detected *in vitro* (Orso et al., 2009; Liu et al., 2012).

The mechanism of ATL-mediated membrane fusion is different from heterotypic fusion, which has been studied extensively using SNARE complex or viral proteins as examples. In those cases, conformation changes of the fusogen, usually helical bundle zippering, drive the fusion reaction. In contrast, the fusion of identical ER membranes, referred to as homotypic fusion, requires ATL to hydrolyze GTP. The ATLs contain an N-terminal GTPase and a three-helix bundle (3HB), followed by two closely spaced transmembrane (TM) segments and a C-terminal tail (CT). The crystal structures of the N-terminal cytosolic domain of human ATL1 (cytATL1) reveal that ATL may adopt three conformations with different nucleotide-bound states (Byrnes et al., 2013). In one conformation the GTPase domains face each other to form a dimer. The 3HBs following the GTPase domains associate with their own GTPase domains and point in opposite directions (Bian et al., 2011; Byrnes and Sondermann, 2011). In another conformation, the GTPase domains remain as a similar dimer, but the 3HBs are parallel to one another and crossover to dock against the GTPase domain of the partner molecule (Bian et al., 2011; Byrnes and Sondermann, 2011). In recent structures, the 3HBs are even closer in the crossed over conformation (Byrnes et al., 2013). These structural data and biochemical studies suggest that dimerization mediated by GTP binding and conformational changes induced by GTP hydrolysis play key roles in the fusion reaction carried out by ATL.

In rodents and higher mammals, each species has three ATL proteins: ATL1, ATL2, and ATL3 (Fig. S1A). Some other species, such as *Drosophila melanogaster* and *Caenorhabditis elegans*, only express one ATL (Rismanchi et al., 2008). Not all eukaryotes possess ATL homologs. A similar class of dynamin-like GTPases has been identified in species lacking ATL, including Sey1p from *Saccharomyces cerevisiae* and Root Hair Defective 3 (RHD3) from *Arabidopsis thaliana* (Hu et al., 2009; Zhang and Hu, 2013). These proteins share similar domain structure and membrane topology with ATL and function in mediating ER fusion (Hu et al., 2009; Anwar et al., 2012; Zhang et al., 2013). Mutations in human ATL1 cause a neurodegenerative disease known as hereditary spastic paraplegia (HSP) (Salinas et al., 2008) and mutations in RHD3 lead to defects in plant growth and short and wavy root hairs (Hu et al., 2009; Zhang and Hu, 2013), suggesting a physiological significance of ER network formation.

Isoforms in the ATL or Sey1p/RHD3 families may coordinate to maintain ER integrity. Arabidopsis expresses two RHD3-like (RL) proteins at a much lower level than RHD3; RL1 is pollen-specific and RL2 is ubiquitous. Individual deletions of these RL proteins cause no detectable defects, suggesting a dominant role of RHD3 (Zhang et al., 2013). However, neither of the RL proteins is dispensable in the background of *rhd3* (Zhang et al., 2013). In mammals, ATL1 is predominantly localized to the central nervous system, whereas ATL2 and ATL3 are expressed in peripheral tissues (Rismanchi et al., 2008). Consistently, almost all HSP-causing mutations are found in ATL1 (Salinas et al., 2008). Whether the ATL family as a whole is essential is not clear. Though ATL2 and ATL3 likely have redundant roles with ATL1, whether these proteins have the same capacity for ER fusion remains to be investigated.

Here, we compare three human ATLs in cells and *in vitro*. We show that ATL3 is a much weaker ER fusogen than ATL1 and ATL2 due to subtle differences in their GTPase activities.

To analyze the functions of ATL2 and ATL3, we used small interference RNA (siRNA) to deplete individual ATL proteins in COS-7 cells and visualized the ER morphology using calreticulin as a marker. The COS-7 cell line is derived from monkey kidney and expresses little ATL1 but sufficient ATL2 and ATL3. When ATL2 was partially knocked down in COS-7 cells, the ER in some cells exhibited unbranched morphology (Fig. 1A–C), indicating defects in ER fusion. However, depletion of ATL3 caused nearly no detectable changes in the ER network (Fig. 1A–C). When cells were treated with siRNAs targeting both ATL2 and ATL3, long unbranched ER tubules were observed in almost all cells (Fig. 1A–C), which is consistent with previous reports (Wu et al., 2014). To rule out the possibility that these results are due to much higher abundance of ATL2, we performed same experiments using HeLa cells, which contain no ATL1 but equivalent amounts of ATL2 and ATL3 (Rismanchi et al., 2008). Even though the ER network in HeLa cells is not very prominent, similar results were obtained as in COS-7 cells (Fig. S2). These findings confirm that ATL activities are required for ER network formation and suggest that ATL2 plays a more dominant role than ATL3 when expressed in the same cells.

**Figure 1:**
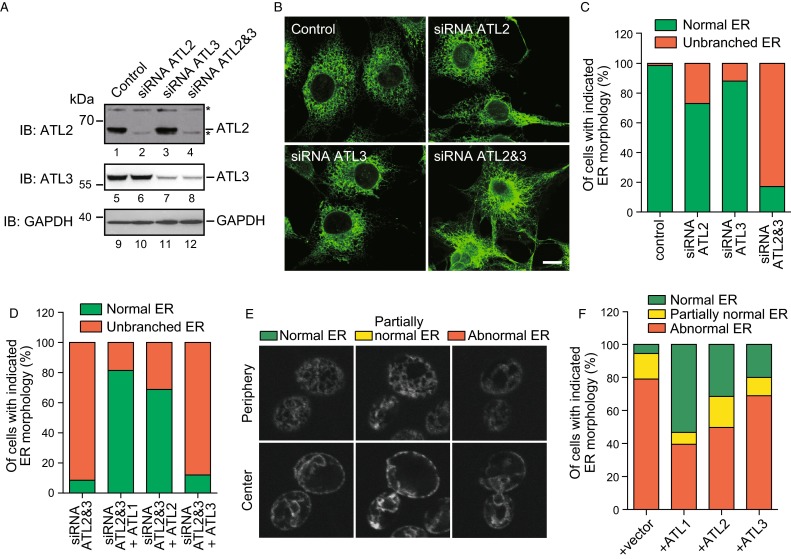
Functional analysis of ATLs in cells. (A) COS-7 cells were transfected with siRNA oligonucleotides as indicated. ATL2 and ATL3 levels were determined by immunoblotting. GAPDH was used as a loading control. Asterisks (*) indicate non-specific bands. (B) The ER morphology of COS-7 cells was visualized using calreticulin, an endogenous luminal ER protein, and indirect immunofluorescence using a confocal microscope. Scale bar = 10 μm. (C) The ER morphology of samples shown in (B) was categorized as “normal” or “unbranched”. A total of 80–150 cells were counted for each sample. All graphs are representative of three repetitions. (D) As in (C), but with samples shown in Fig. S3A. (E) The ER morphology in yeast cells expressing the GFP fusion ER protein Sec63p was categorized as “normal”, “partially normal”, or “abnormal”. Representative views of each category were shown by confocal microscopy focusing at the center or periphery of the cells. Scale bar = 1 μm. (F) Empty vector or indicated ATLs were expressed with a CEN plasmid under the control of the endogenous SEY1 promoter in *sey1Δyop1Δ* cells. The ER morphology was determined as in (E). At least 100 cells were counted for each sample. The results are representative of at least two repetitions

To compare the abilities of all three ATLs to mediate ER fusion, COS-7 cells depleted of both ATL2 and ATL3 were transfected with each ATL protein and the ER morphology visualized (Fig. S3A). As expected, ATL1 expression efficiently restored the ER network in double-depleted cells (Fig. 1D). On the other hand, expression of ATL2 only partially rescued the ER morphology, and a significant number of cells still contain unbranched ER tubules when ATL3 was expressed (Fig. 1D). These results suggest that the three ATLs have different capacities to mediate ER fusion, with ATL1 being the strongest and ATL3 being the weakest.

To confirm the results from COS-7 cells, we tested whether three ATLs can replace Sey1p in *S. cerevisiae* to a similar extent. Yeast cells lacking Sey1p and one of the two proteins that shape the ER tubules (Yop1p or Rtn1p) exhibited abnormal cortical ER morphology; in particular, the tubular ER network largely disappeared and many areas of the cortex were void of ER, indicating a lack of ER fusion (Hu et al., 2009). These defects can be restored by the expression of wild-type Sey1p or human ATL1 using a CEN vector with the endogenous *SEY1* promoter (Hu et al., 2009; Anwar et al., 2012). To better evaluate ER morphology, which is visualized by Sec63p-GFP, we added a category of “partially normal ER” in which some fenestrated ER was observed but areas of cortex lacked ER (Fig. 1E). Consistently, the expression of ATL1 in *sey1Δyop1Δ* cells rescued the ER morphology (Figs. S3B and 1F). In contrast, ATL3 exhibited a poor ability to replace Sey1p in yeast (Fig. 1F), even if expressed at a much higher level than ATL1 (Fig. S3B). The expression of ATL2 was not comparable to that of ATL1 and ATL3 in yeast cells (Fig. S3B), even though the same promoter was used. Nevertheless, some improvements in ER morphology were observed when ATL2 was transformed into *sey1Δyop1Δ* cells (Fig. 1F). Taken together, the results indicate that ATL3 behaves as a much weaker ER fusogen in mammalian and yeast cells than ATL1 and ATL2.

Previous studies have shown that ATL-mediated fusion is initiated by GTP-dependent dimerization between ATL molecules in apposing membranes, and the reaction proceeds when GTP hydrolysis drives conformational changes in ATL. To test whether the varied fusion abilities of the three ATLs are caused by different dimerization tendencies of ATLs upon GTP binding, we compared the dimerization state of purified cytATLs using analytic ultra-centrifugation (AUC). At low concentrations (12 μmol/L), cytATL1 formed some dimer in the presence of GMP-PNP, a non-hydrolyzable analog of GTP (Fig. 2A). The proportion of dimer was increased in the presence of a higher concentration of cytATL1 (24 μmol/L, Fig. 2A). Previously, cytATL1 was shown to behave as a dimer when an even higher protein concentration (50 μmol/L) was used in AUC (Bian et al., 2011). CytATL2 exhibited similar GTP-induced dimerization as ATL1 (Fig. 2B), but little dimer was seen when ATL3 was tested at the same concentrations (Fig. 2C).

**Figure 2:**
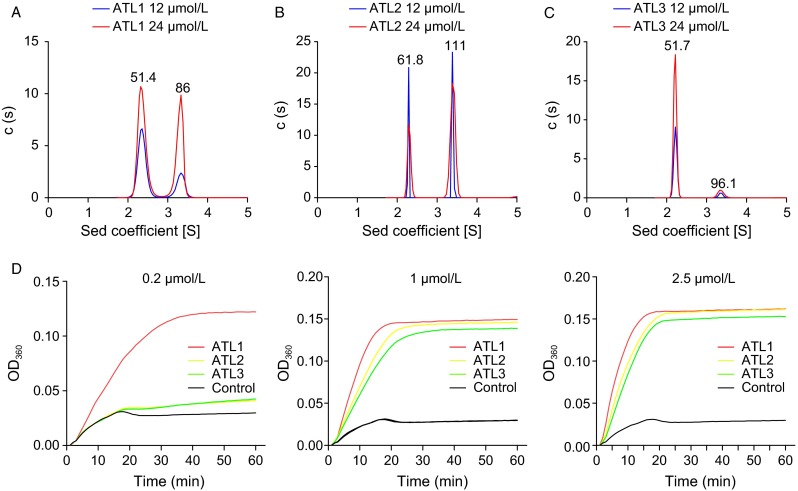
Biochemical comparison of cytATLs. Nucleotide-dependent dimerization of (A) cytATL1, (B) cytATL2, and (C) cytATL3 (12 μmol/L shown in blue, 24 μmol/L shown in red) were determined by analytical ultracentrifugation in the presence of GMP-PNP. The estimated molecular masses are given above the peaks (in kDa). (D) The GTPase activities of the N-terminal cytosolic domains of wt ATL1, ATL2, and ATL3 (0.2 μmol/L, 1 μmol/L, and 2.5 μmol/L) were measured by phosphate release at saturating concentrations of GTP (0.5 mmol/L). All graphs are representative of three repetitions

Finally, we compared the GTPase activities of purified cytATLs. When the GTPase assay was performed using cytATL at a concentration of 2.5 μmol/L, similar rates of GTP hydrolysis were detected (Figs. 2D and S3C). However, the GTPase activities of ATL2 and ATL3 were significantly lower than that of ATL1 at low protein concentrations (Figs. 2D and S3C). Taken together, these results suggest that peripheral ATLs, especially ATL3, are considerably weaker than ATL1 in GTP hydrolysis, likely due to a lower propensity for GTP-dependent dimerization.

Our results reveal that three human ATLs mediate ER fusion at different capacities: ATL1 is the strongest and ATL3 is the weakest. The primary structures of these dynamin-like GTPases are highly conserved, with more than 60% sequence identity (Rismanchi et al., 2008). Almost all key residues for ATL dimerization, GTP hydrolysis and conformational changes, identified through structural and biochemical analysis of ATL1, can be found in ATL2 and ATL3 (Bian et al., 2011). Thus, it was surprising that the ATLs behaved differently, in both mammalian cells and yeast cells, in regard to maintaining proper ER morphology. When we analyzed the difference using purified cytATLs, the ability of ATLs to mediate ER fusion correlated with their GTPase activity, likely due to subtle differences in nucleotide-dependent dimerization. Therefore, we speculate that the residues supporting the key properties of ATLs, particularly for dimer formation, may be different. In addition, small differences in GTPase activity may be amplified during ER fusion in cells.

In humans, ATL1 is expressed predominantly in the central nervous system. Conversely, ATL2 and ATL3 are abundant in peripheral tissues. Some tissues, such as skeletal muscle, are enriched with ATL2, and others, such as the lung, contain more ATL3 (Rismanchi et al., 2008). The tissue distribution explains why almost all HSP-causing mutations are found in ATL1, given that HSP is a degenerative neuron disease. Notably, low levels of ATL3 are also found in brain tissue, which is consistent with a recent description of a rare case in which ATL3 mutation caused hereditary sensory neuropathy (Kornak et al., 2014). This case implies that, even as a weak force for ER fusion, ATL3 can still be critical for the physiological balance of the ER. Reminiscent examples include RL proteins auxiliary to the mediation of plant ER fusion and essential when RHD3 is missing.

Given the weak activity of ATL3-mediated ER fusion, ER fusion likely occurs much less frequently in tissues primarily expressing ATL3. In contrast, in cells with long protrusions, such as nerve cells with axons or plant cells with root hair, the importance of ER connectivity is greater than usual, and the dominant form of the ER fusogen in the corresponding species, i.e. human ATL1 and Arabidopsis RHD3, is expressed at a very high level. Therefore, the level of ER fusion is likely tightly regulated by the expression pattern of the three ATLs in response to cell type-specific cellular functions, and may even involve crosstalk between different ATLs. Another dynamin-like GTPase family in mammals, called mitofusin (MFN), also mediates homotypic membrane fusion (Hoppins et al., 2007), in this case in outer mitochondrial membranes, and has the same molecular architect and membrane topology as ATLs. The MFN family has two members, MFN1 and MFN2. Deletion of either protein causes mitochondrial fragmentation due to a lack of fusion. MFN1 and MFN2 have different GTPase activities and may function in distinct physiological pathways (Ishihara et al., 2004). Thus, differential fusogenic capacity among family members may be a common way of regulating organellar dynamics in different cells.

## Footnotes

We thank Wendan Chu and Zheng Wang for technical assistance. This work was supported by National Natural Science Foundation of China (Grant No. 31225006), and an International Early Career Scientist grant from the Howard Hughes Medical Institute.

Xiaoyu Hu, Fuyun Wu, Sha Sun, Wenying Yu, and Junjie Hu declare that they have no conflict of interest. This article does not contain any studies with human or animal subjects performed by the any of the authors.

## Electronic supplementary material

Below is the link to the electronic supplementary material.

Supplementary material 1 (PDF 4941 kb)
