# Supplementary material for: Human atlastin GTPases mediate differentiated fusion of endoplasmic reticulum membranes
Source: Protein Cell. 2015 Mar 17;6(4):307–11. doi: 10.1007/s13238-015-0139-3 (PMC4383757; doi:10.1007/s13238-015-0139-3)
Supplement: Supplementary file 1 — Supplementary material 1 (PDF 4941 kb) [file 13238_2015_139_MOESM1_ESM.pdf]

Electronic supplementary material

Materials and Methods

Molecular cloning and antibodies

For expression in mammalian cells, the pGW1 constructs expressing Myc-tagged human ATL1, ATL2, and ATL3 were described previously (Zhu et al., 2003). siRNA-resistant ATL constructs were generated using the QuikChange Site-Directed Mutagenesis Kit (Stratagene). For expression in yeast, ATL2 or ATL3 with an N-terminal HA tag were subcloned into pRS316 (a URA3/CEN plasmid) with the endogenous promoter and terminator of SEY1 as described previously for ATL1 (Anwar et al., 2012). For expression in *E. coli*, the N-terminal cytosolic domains of ATL2 and ATL3 were subcloned into the pET28 vector as described previously for ATL1 (Bian et al., 2011). All constructs were verified by DNA sequencing. Anti-HA antibodies were from Roche, anti-calreticulin antibodies from Abcam, anti-phosphoglycerate kinase (PGK) antibodies from Invitrogen, and anti-Myc antibodies from Santa Cruz.

Mammalian cell culture and transfection

COS-7 cells (American Type Culture Collection) were maintained in complete Dulbecco's minimum essential medium supplemented with 10% fetal bovine serum in 5% CO_2 at 37°C. Transfections were performed using Turbo (Thermo) or Lipofectamine 3000 according to the manufacturer's instructions. siRNA studies for ATL2 and ATL3 in COS-7 cells were performed as described previously (Rismanchi et al., 2008), except that siRNA transfections were performed using RNAi max (Invitrogen). For rescue experiments, cells were transfected with siRNA-resistant ATL constructs after 24h of siRNA treatment.

Immunofluorescence and confocal microscopy

Indirect immunofluorescence of paraformaldehyde-fixed cells was described previously (Hu et al., 2009). Transfected cells were grown on coverslips and immunostained with anti-calreticulin (abcam; 1:500) and anti-Myc antibodies (abcam; 1:500) as primary antibodies and various Alexa Fluor-conjugated secondary antibodies (AlexaFluor 488 anti-mouse or AlexaFluor 568 anti-rabbit, 1:1000, Invitrogen). Yeast cells were imaged live as described previously (Hu et al., 2009). All images were captured on an OLYMPUS FV1200 confocal microscope with a 60×/1.40 NA Plan Apochromat oil immersion objective lens using the Olympus Fluoview Version 2.0b Viewer. Brightness and contrast were adjusted across the entire image using Adobe Photoshop.

Protein purification

The N-terminal cytosolic domains of human ATL1 (residues 18–447), ATL2 (residues 1-474), or ATL3 (residues 1-443) with N-terminal, thrombin-cleavable His6-tags were expressed in *E. coli* and purified as described previously. Briefly, the proteins were isolated by Ni-NTA chromatography and further purified by ion-exchange chromatography and gel filtration.

Analytical ultracentrifugation

Sedimentation velocity experiments were performed in a ProteomeLab XL-1 Protein Characterization System (Beckman Coulter). All interference data were collected at a speed of 201,600 × g in an An-50 Ti rotor at 4°C. A set of 999 scans was collected at 4-min intervals. ATL1, ATL2, or ATL3 proteins were prepared in a buffer containing 50 mM Tris HCl (pH 8.0), 5 mM $MgCl_2$, and 1 mM DTT at 0.6 mg/ml or 1.2 mg/ml. Data were analyzed by the program SEDFIT (version 11.8) in terms of a continuous c(s) distribution.

GTPase activity assay

GTPase activities were measured with the Enzchek phosphate assay kit (Invitrogen). Reactions were performed in a 200 µl volume with 10 µl of 20× reaction buffer (1 M Tris HCl/20 mM $MgCl_2$, pH 7.5/2 mM sodium azide), 200 mM 2-amino-6-mercapto-7-methylpurine riboside, 1 unit/ml purine nucleoside phosphorylase (PNP), and 0–2.5 µM ATL1, ATL2, and ATL3 protein, and then incubated for 30 min at 37°C in a 96-well plate (Thermo). Reactions were initiated by the addition of 0.5 mM GTP. The absorbance at 360 nm was measured every 1 min over 60 min at 37°C using a microplate reader (TECAN infinite M200 PRO).

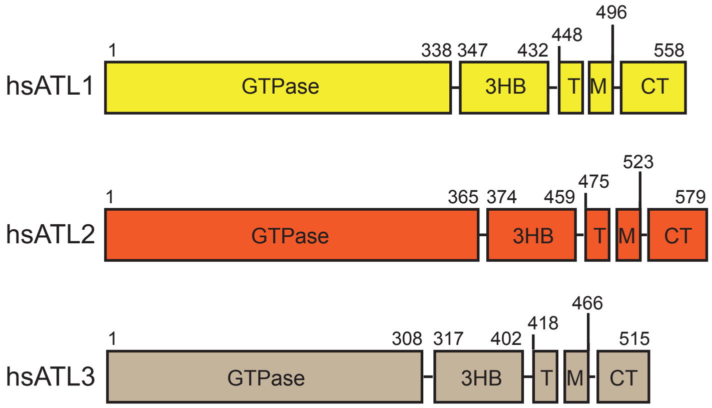

Supplementary Figure 1. Domain structures of human ATLs.
Scheme showing the domains of human ATL1, ATL2 and ATL3. 3HB, three-helix bundle; TMs, transmembrane segments; CT, C-terminal tail. The residue numbers of each domain are listed.

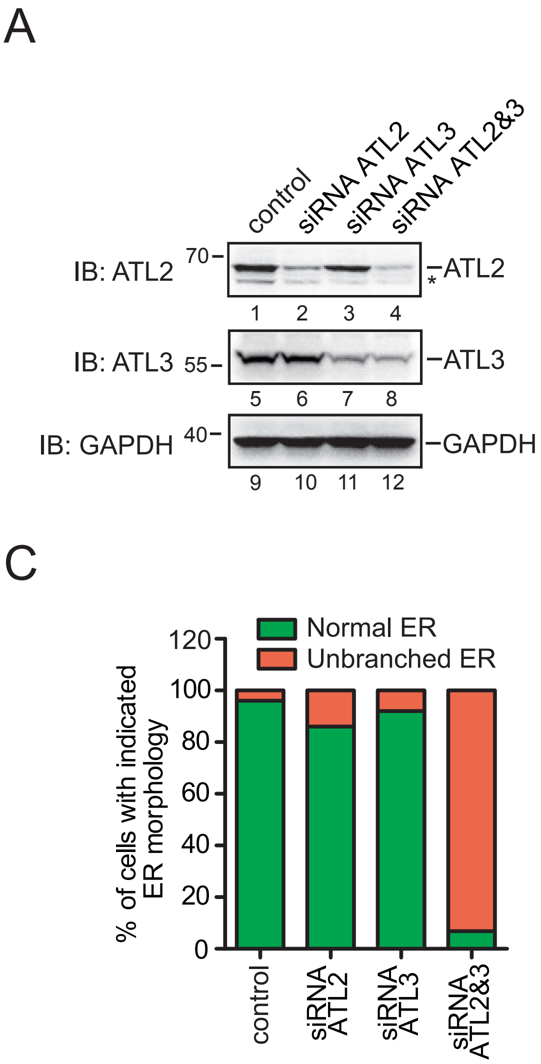

Supplementary Figure 2. Functional analysis of ATLs in Hela cells.
(A) Hela cells were transfected with siRNA oligonucleotides as indicated. ATL2 and ATL3 levels were determined by immunoblotting. GAPDH was used as a loading control. Asterisk (*) indicates a nonspecific band. (B) The ER morphology of Hela cells was visualized using calreticulin, an endogenous luminal ER protein, and indirect immunofluorescence using a confocal microscope. Scale bar = 10 μm. (C) The ER morphology of samples shown in B was categorized as "normal" or "unbranched". A total of 80-150 cells were counted for each sample. All graphs are representative of three repetitions.

.

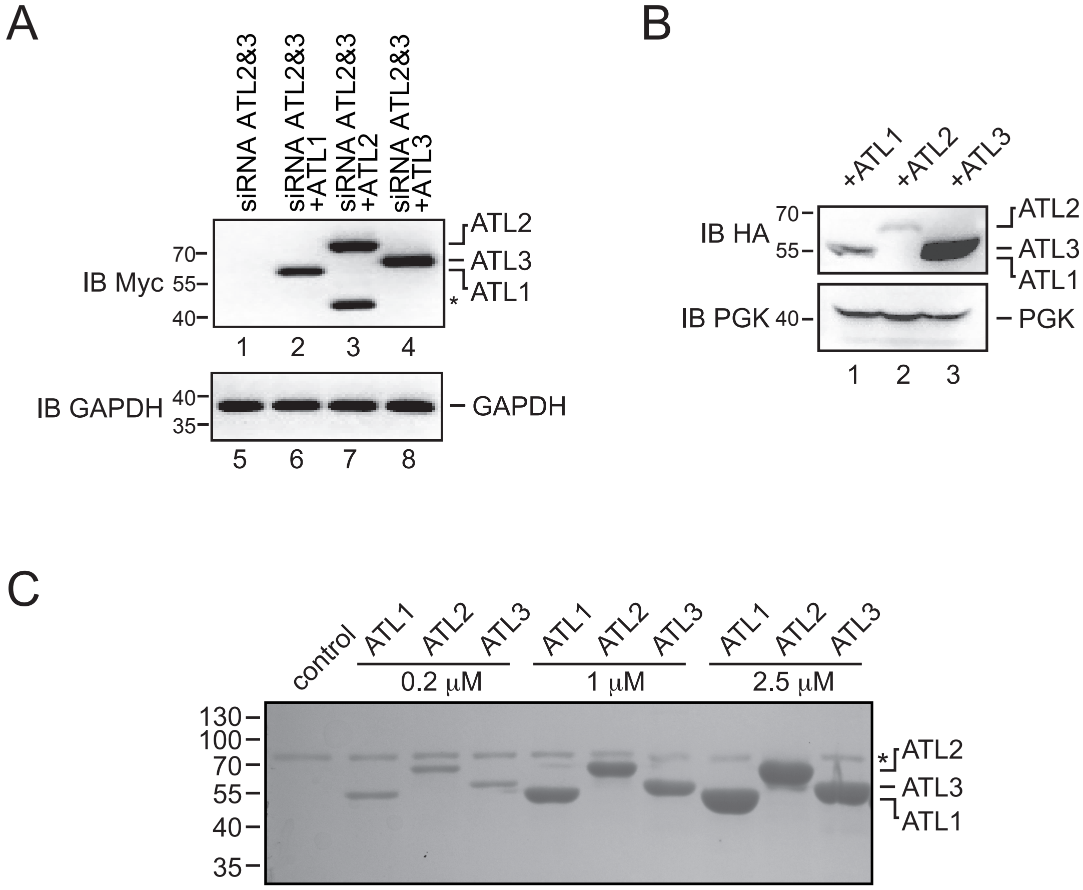

Supplementary Figure 3. Protein levels in the assays shown in Fig. 1 and Fig. 2

(A) COS-7 cells used in Fig. 1D were double-depleted for ATL2 and ATL3, and then transfected with Myc-ATL1, 2, or 3. Myc-ATLs levels were determined by immunoblotting. Asterisks (*) indicate degradation products. GAPDH was used as a loading control. (B) The expression levels of ATL1, ATL2, and ATL3 in yeast cells used in Fig. 1F were determined by immunoblotting. PGK was used as a loading control. (C) The levels of ATL1, ATL2, and ATL3 used in Fig. 2D were analyzed by SDS-PAGE and Coomassie blue staining. The asterisk (*) indicates the PNP protein used in the GTPase assay.
